# Comparative Analysis of Metal Binding Characteristics of Copper Chaperone Proteins, Atx1 and ATOX1

**DOI:** 10.1155/S1565363304000081

**Published:** 2004

**Authors:** Suree Narindrasorasak, Xuefeng Zhang, Eve A. Roberts, Bibudhendra Sarkar

**Affiliations:** 1 From the Department of Structural Biology and Biochemistry The Research Institute of the Hospital for Sick Children Toronto Ontario M5G 1X8 Ontario Canada; 2 Permanent address:Metabolism Research Program The Research Institute of The Hospital for Sick Children and Department of Pediatrics Medicine and Pharmacology University of Toronto, Toronto Ontario Canada; 3 The Department of Biochemistry University of Toronto Toronto Ontario M5S 1A8 Canada

## Abstract

The metal binding properties of the human copper chaperone ATOXI and its yeast homologue Atxl have been characterized. Complexes of these proteins with Cu(I), Ag (1), Cd(II) and Hg(II) were studied by native gel electrophoresis, chemical cross-linking followed by SDS-PAGE, as well as by size exclusion chromatography, mutagenesis and UV-visible absorption spectroscopy. Results indicate that binding of different metals to either ATOXI or Atxl altered conformation of subunit structure and the oligomerization state of the proteins. Furthermore, it has been demonstrated that freshly reduced apoprotein is capable to convert Cu(ll) to Cu(l) stoichiometrically to the amount of protein present, while oxidized protein is only twenty per cent as active. Titration of Cu(ll) with either oxidized or reduced protein resulted in similar increase in absorbance at 254 nm, implicating Cu-thiolate formation in both forms of the protein, but titration with Ag(i) caused the increase in absorbance at 254 nm with the reduced protein only. These data indicate that Cu(1), Ag(1), Hg(ll) and Cd(ll) are all capable of binding to ATOXI and Atxl, but the characteristics of the binding to these copper chaperones differ for different metals.


### Comparative Analysis of Metal Binding Characteristics of Copper Chaperone Proteins, Atx1 and ATOX1[†]

Suree Narindrasorasak[‡], Xuefeng Zhang[‡], Eve A. Roberts[§], and Bibudhendra Sarkar[‡¶,*]

[‡]*From the Department of Structural Biology and Biochemistry, The Research Institute of the Hospital for Sick Children, Toronto, Ontario, M5G 1X8 Ontario Canada and* [¶]*The Department of Biochemistry, University of Toronto, Toronto, Ontario, M5S 1A8 Canada.*

[§] *Permanent address: Metabolism Research Program, The Research Institute of The Hospital for Sick Children and Department of Pediatrics, Medicine and Pharmacology, University of Toronto, Toronto, Ontario, Canada.*

### ABSTRACT

The metal binding properties of the human copper chaperone ATOX1 and its yeast homologue Atx1 have been characterized. Complexes of these proteins with Cu(I), Ag (I), Cd(II) and Hg(II) were studied by native gel electrophoresis, chemical cross-linking followed by SDS-PAGE, as well as by size exclusion chromatography, mutagenesis and UV-visible absorption spectroscopy. Results indicate that binding of different metals to either ATOX1 or Atx1 altered conformation of subunit structure and the oligomerization state of the proteins. Furthermore, it has been demonstrated that freshly reduced apoprotein is capable to convert Cu(II) to Cu(I) stoichiometrically to the amount of protein present, while oxidized protein is only twenty per cent as active. Titration of Cu(II) with either oxidized or reduced protein resulted in similar increase in absorbance at 254 nm, implicating Cu-thiolate formation in both forms of the protein, but titration with Ag(I) caused the increase in absorbance at 254 nm with the reduced protein only. These data indicate that Cu(I), Ag(I), Hg(II) and Cd(II) are all capable of binding to ATOX1 and Atx1, but the characteristics of the binding to these copper chaperones differ for different metals.

[1]**Abbreviations:** BCA, bicinchoninic acid; BCS, bathocuproinedisulfonic acid; BSA, bovine serum albumin; DTT, dithiothreitol; EDTA, ethylene diamine tetra-acetic acid; GST, glutathione S-transferase; HEPES, N-(2-hydroxyethyl)piperazine-N′-2-ethanesulfonic acid, IPTG; isopropyl-β-D-thiogalactopyranoside; MES, 2-

---

[†] This work is supported by a grant from the Canadian Institutes of Health Research MOP 1800 to B.S.

[*] To whom correspondence should be addressed: Department of Structural Biology and Biochemistry, The Hospital for Sick Children, 555 University Ave., Toronto, Ontario, M5G 1X8 Canada. Tel: 416-813-5921, Fax: 416-813-5022, email: bsarkar@sickkids.on.ca

(N-morpholino)ethanesulfonic acid; PAR, 4-(2-pyridylazo)resorcinol; PMSF, phenylmethylsulfonyl fluoride; SDS-PAGE, SDS polyacrylamide gel electrophoresis; Tris, tris(hydroxymethyl)amminomethane.

### INTRODUCTION

Copper is an essential trace element in all living organisms. It plays a crucial role in cellular respiration, antioxidant defense and iron metabolism in eukaryotes /1/. Since free intracellular copper can be toxic even at low concentrations, specialized systems for transport and homeostatic mechanisms are required to maintain safe intracellular concentrations of this metal inside the cells. Insight into the mechanism of copper trafficking has followed the characterization of genes involved in the inherited copper disorders, Wilson and Menkes diseases /2-5/. Despite strikingly different clinical phenotypes, each disease results from absence or dysfunction of homologous copper transporting ATPases located in the trans-Golgi network of cells for transporting copper to the secretory pathway and cellular export /6-10/. In Wilson disease, toxic amounts of copper accumulate in liver and brain, resulting in hepatic cirrhosis and neuronal degeneration. Menkes syndrome is characterized by a profound copper deficiency state which results in the failure to incorporate copper into essential copper containing enzymes /11/. Studies on a similar ATPase, Ccc2p in *Saccharomyces cerevisiae* have revealed a remarkable conservation of mechanisms of copper metabolism /12/.

A series of genetic studies in *Saccharomyces cerevisiae* has revealed that the delivery of copper to specific cellular pathways is mediated by a group of proteins called copper chaperones. One of these chaperones in yeast is Atx1 /13/, a small molecular weight protein which contains a single repeat of the MXCXXC copper-binding motif present in the Wilson and Menkes proteins (each of which contains six repeats of this motif /14/) and functions to deliver copper to the secretory pathway for biosynthesis of Fet3p, a multicopper oxidase homologous to ceruloplasmin, required for high-affinity iron uptake in yeast /15/. This MXCXXC motif is also found in the copper chaperone (CCS) for superoxide dismutase I (SOD1) /16/, bacterial copper chaperone CopZ /17/, and in a variety of bacterial metal-transport proteins, including ZntA /18/, CopA /19/, the Hg(II)-resistance protein MerP and other proteins involved in resistance to Cd(II) and Pb(II) /14,20/. Atx1 interacts specifically with the yeast homologue of the Menkes and Wilson disease proteins, Ccc2p, and transfers copper to the N-terminal cytosolic domain of Ccc2p, which contains two repeats of metal binding motif /21,22/.

ATOX1 (or HAH1) has been identified as a human homologue of Atx1 /23/. This protein has been shown to complement functionally *atx1Δ* mutant strains in yeast, suggesting that ATOX1 plays a role in copper homeostasis in mammalian cells. Recently, similar proteins have been identified in rats /24/, dogs /25/, sheep /26/ as well as in mice /27/. Both the Menkes and Wilson disease proteins are thought to acquire copper via ATOX1 /23,28/. It has been shown that ATOX1 interacts with Menkes and Wilson proteins in a copper dependent fashion /29,30/; however, the molecular events involved in copper transfer remain poorly understood.

X-ray absorption spectroscopic studies indicate that Menkes and Wilson proteins bind Cu (I) as two-coordinate via two Cys residues in the MXCXXC motif /31,32/, whereas a third ligand, probably an exogenous thiol, is observed in Atx1 /21/. A high resolution X-ray structure of Hg(II)-Atx1 reveals that the mercury is coordinated in a bidentate fashion from two cysteine sulfurs with a S-Hg bond angle of 167° /33/. The NMR structures of apo- and copper bound Atx1 have also been reported /34/. In both cases, the metal bound Atx1 exists as monomer. The Atx1 metallochaperone /33/, the fourth metal binding domain of Menkes protein /35/, the bacterial copper binding protein CopZ in *E. hirae* /36/, and *B. subtilis* /37/, as well as the bacterial periplasmic protein MerP /38/ all adopt a βαββαβ structural fold and exhibit one metal bound per molecule of protein. X-ray crystal structures of Hg(II), Cd(II) and Cu(I)-bound form of ATOX1 also exhibit βαββαβ fold but, unlike the aforementioned proteins, two adjacent molecules of ATOX1 are linked by a metal ion /39/. In Cd(II)-ATOX1, the Cd(II) ion is coordinated in a tetrahedral fashion by four Cys residues, similar to those observed in the structure of Cd(II)5Zn(II)2 form of rat metallothionein. In Cu(I)-ATOX1 and Hg(II)-ATOX1, the metal binding site exhibits distorted tetrahedral geometry. Structural studies of ATOX1 suggest the molecular concept of how metal ion transfer between MXCXXC containing domains takes place. The two MXCXXC motifs can dock with one another in a manner that supports the direct metal transfer mechanism proposed by Pufahl *et al.* /21/.

Despite the similarity between Atx1 and ATOX1, to date there have been no studies examining the dimerized state of metal-bound Atx1. This report presents evidence that both Atx1 and ATOX1 exist as a mixture of monomers, and dimers, and that different metals or redox states confer different conformations and oligomerization of both proteins.

### MATERIALS AND METHODS

### Cloning of ATOX1 and Atx1

Both ATOX1 and Atx1 were expressed as fusion proteins with GST (glutathione-S-transferase) at the N-terminal portion of the molecule, using expression vector pGEX-6P-2 (Amersham-Pharmacia Biotech), according to standard protocols of molecular biology. The vector contains a "Prescission" protease (human rhinovirus 3C protease) cleavage site, enabling the removal of GST moiety from the cloned proteins at the final step of purification.

### Site directed mutageneses of ATOX1

Mutageneses on the construct of ATOX1 in pGEX-6P-2 vector were performed by using a USE Mutageneses Kit (Amersham Pharmacia Biotech) as per protocol. Five mutants of ATOX1 with Cys residues mutated to Ser in various combination were generated, namely, C41S-ATOX1 (where $C^{41}$, which is not part of metal binding motif was mutated to S), CC/SS-ATOX1 (both conserved Cys residues, $C^{12}$ and $C^{15}$ were mutated to S ), 3C/3S-ATOX1(all 3 Cys in ATOX1 were mutated), CC/SC-ATOX1 (only $C^{12}$ was mutated) and CC/CS-ATOX1 (only $C^{15}$ was mutated). The fidelity of the constructs was verified by automated DNA

sequencing (DNA Sequencing Facility, Center for Applied Genomics, Hospital for Sick Children, Toronto, Canada).

### Expression and purification of Atx1, ATOX1 and its mutants

All of the constructs generated as described above were used to transform *E. coli* BL21 (DE3) cells for protein expression. The fusion proteins were present mainly in soluble forms. The purification of GST-fusion proteins, and subsequent removal of GST, were essentially as described previously /31/, but without urea in the buffers. The final yield of purified ATOX1 (or its mutants, as well as Atx1) was between 50-80 mg per 5 L of *E. coli* culture. Protein concentration was determined by BCA method (Pierce) using BSA as standard. The final protein products contained no bound metal.

### Size exclusion chromatography of the metal-protein complexes

The metal protein complexes of ATOX1 or Atx1 were prepared by incubating purified protein (0.1 - 1 mM) in 0.5 ml of 20 mM MES pH 6.0, containing one molar equivalent of metal (Cu (II),or Ag(I), or Cd(II)), and 3 molar equivalent of DTT, at room temperature for 30 min. The excess metal and reducing agent were removed by gel filtration on Bio-Gel P-10 (Bio-Rad) column (separation range 20 kD-1 kD), with the column dimension of 1.5 x 60 cm. One-ml fractions were collected. The molecular sizes of the eluted metal-protein complexes were assessed by comparison with elution profiles of molecular weight standard proteins, applied to the same column.

### Determination of Free Thiols (Ellman's Assay)

The free thiol group was determined in the presence and absence of 6 M guanidine.HCl in 0,1 M phosphate buffer pH 8.0, with addition of 0.1 mM DTNB (dithio-1,4-nitrobenzoic acid). The change in absorbance at 412 nm was monitored at 25°C and the total sulfhydryl content was calculated according to the method described by Creighton /40/.

### Determination of metal content in metal protein complexes

The extent of copper, or cadmium incorporation was determined by spectrophotometry, using the metallochromic indicator 4-(2 pyridylazo)-resorcinol (PAR) to determine the amount of metal released after treatment of metal-protein complexes with sodium hypochlorite (NaOCl), as described by Casadevall and Sarkar /41/ with some modifications. Copper(I) content in copper bound protein was also determined by BCA reaction described by Brenner and Harris /42/. Alternatively, the Cu(I) content in the mixture was measured by the BCS competition reaction. This reagent was also used to determine Cu(I) in the presence of Cu(II) directly in titration experiments described in the following section. The appearance of a Cu(BCS)2

complex was measured by monitoring the absorbance at 483 nm, with a molar extinction coefficient of 12250 /43/.

### UV-visible spectra of metal-protein complexes

All UV-visible spectra were recorded on a Hitachi U-3210 Spectrophotometer. Spectra of metal-protein complexes were generated by direct titration of aliquots of Cu(II) or Ag(I) solution with freshly reduced proteins (prepared by incubation of the proteins with DTT followed by gel filtration to remove excess DTT), or oxidized proteins (proteins that had been stored at 4 °C for at least 2 weeks). The spectra between wavelengths of 240-360 nm were recorded; the metal-complex formations were followed by measuring increases in absorbance at 254 nm.

### Chemical Cross-Linking

Cross-linking reactions were carried out in the absence and presence of metal ions, according to the manufacturer's protocol. The cross-linking agents used were DMS (dimethylsuberimidate (Pierce), spacer arm 11 Å) and EGS (ethylene glycobis(sulfosuccunimidylsuccinate (Pierce), spacer arm 16.1 Å). Both are reactive toward amino acid containing primary amine groups such as Lys residues. The cross-linked products were analysed on SDS-PAGE. Control reactions, without cross-linkers, were performed in parallel.

### RESULTS AND DISCUSSION

### Gel mobility patterns of different metal bound forms of ATOX1 on native and SDS gel electrophoresis.

Figure 1A shows mobility patterns on native-PAGE of apo (untreated), reduced, and metal-bound ATOX1 in the presence of mild reducing condition (reducing agent was used at equimolar concentration of Cys-residues in the protein). The untreated apo-ATOX1 remained close to the top of the gel. In the presence of DTT, the apoprotein migrated faster toward anode and seemed to consist of more than one species. The Cu-complex of ATOX1 only moved slightly faster than untreated apo, followed by Ag(I)-, Hg(II)- and Cd(II)-ATOX1 which migrated the farthest. Mobility of proteins on a native gel electrophoresis depends on various factors, including conformation, size and overall charge of the proteins. Thus the different migration patterns displayed by these metal-ATOX1 complexes suggest that these metals affect the conformation or subunit structure of the protein differently.

An attempt to identify the effects of these metals on subunit structure of ATOX1 by using SDS-PAGE did not provide any conclusive information, as shown in Figures 1B and 1C. When these complexes were separated on an SDS gel in the absence of reducing agent in gel sample buffer, the untreated apoprotein showed the presence of some dimer as well as trimer. All of the metal complexes also had the similar degree of dimerization; however, the Cu(I)-ATOX1 showed a band smearing upward from monomer toward the dimer position (Figure 1B). This band was reversible since it disappeared when SDS gel was performed

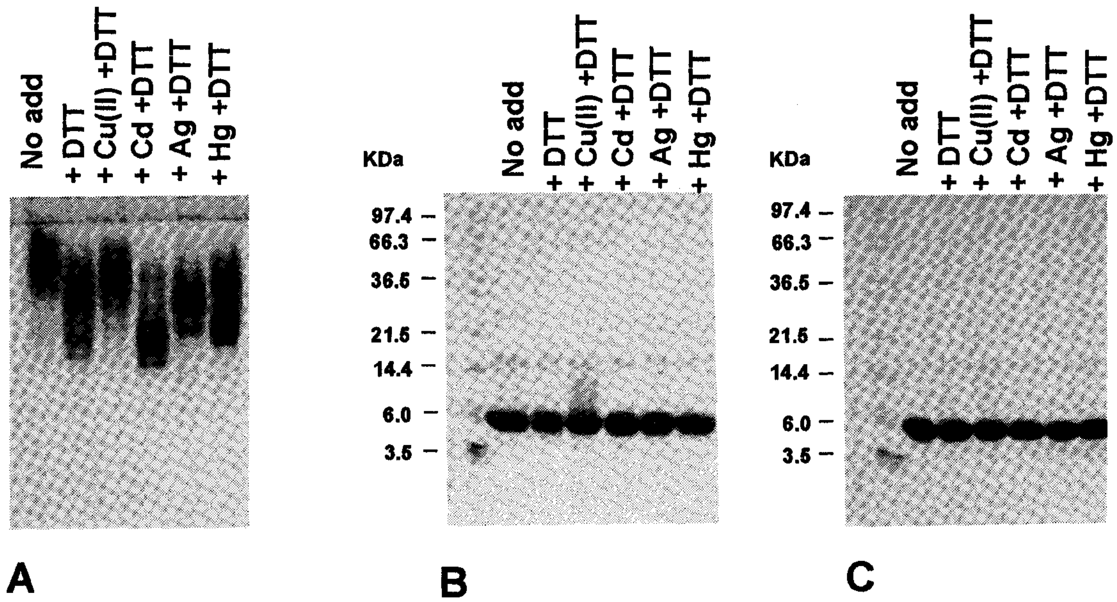

**Fig. 1:** Gel migration pattern of ATOX1 on 10% Native-PAGE (A) and 16% SDS-PAGE in the absence (B) or presence of 300 mM 2-mercaptoethanol (C). Ten μl of 0.3mM of untreated, reduced (+DTT, 3:1 molar ratio) or 1:1 metal/protein + DTT was loaded on the gel as indicated on each lane. No add = apoprotein without DTT or metal.

using sample buffer containing excess reducing agent, as shown in Figure 1C. Results from SDS gels also indicated that some of the dimer bands observed on the gel were not formed through disulfide bonds, as the electrophoretic patterns remained even in the presence of reducing agent. Results obtained from native gel (Figure 1A) initially led us to suspect that the untreated (oxidized) apo-ATOX1 might exist as an oligomer whereas the fully reduced apo-ATOX1, as well as Cd(II)-complex, is a monomer, and Cu(I)- or Ag(I)-complex is a dimer. Failure to detect increased oligomerization on SDS gel might be due to the instability of dimerization by metal such that in the presence of the strong negative charge of SDS the complex was able to dissociate.

One approach to capture different stages of oligomerization of protein is to use a cross-linking reagent. Thus cross-linking reactions between metal-ATOX1 complexes and DMS, or EGS were performed, and the products were separated on SDS gel. Contrary to our suspicion that the Cd(II)-ATOX1 complex may exist as a monomer, results shown in Figure 2A and 2B indicated that out of the four metal-complexes, Cd(II)-ATOX1 could be cross-linked with EGS as a dimer. Dimerization of Cd(II)-ATOX1 could also be captured by using DMS, but to a much lesser extent. Hg(II)-ATOX1 complex could also form a dimer, though to a lesser extent than Cd(II)-ATOX1. Furthermore, faint bands of trimers and tetramers are also detected in the cross-linked products of metal complexes. Results obtained from these cross-linking experiments implied that Cd(II) binding to ATOX1 altered the conformation on the protein so that Lys residues from each subunit became close enough to facilitate cross-linking reactions. This result also helps explain the behavior of migration of Cd(II)-ATOX1 complex on native-PAGE. On native-PAGE, protein containing more exposed basic residues migrates slower toward cathode. Binding of Cd(II) to ATOX1 may change conformation of the

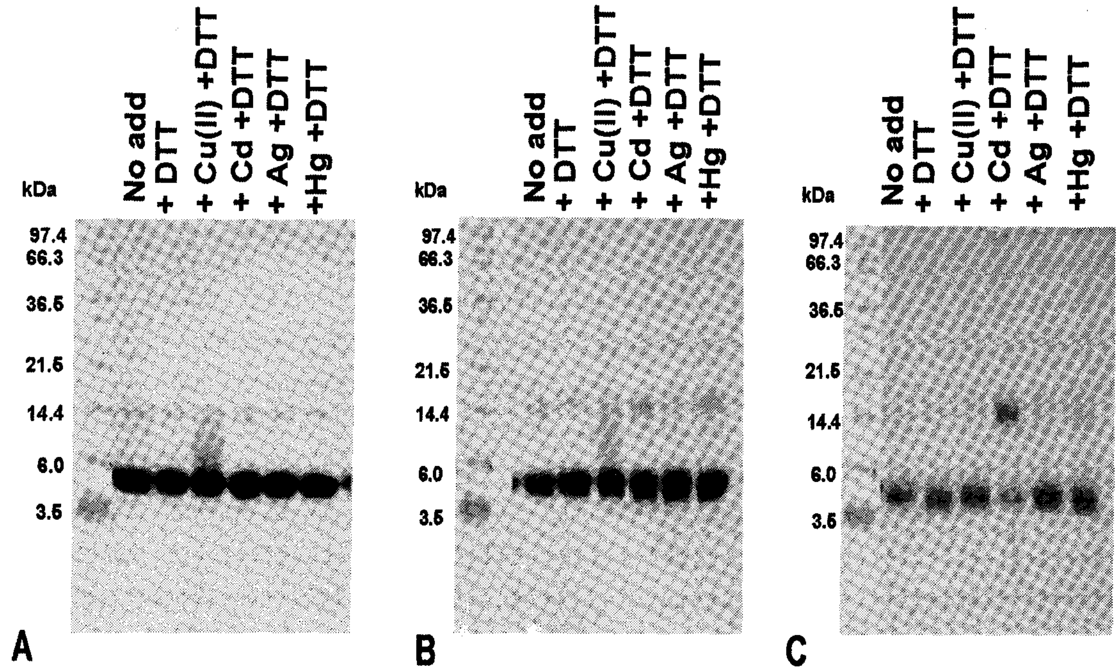

**Fig. 2:** SDS-PAGE of crosslinked products of ATOX1 with DMS or EGS in the absence and presence of metals + DTT. Cross-linking reactions were performed as described in the Materials and Methods section. Ten μl of cross-linked products were loaded on the gel as indicated on each lane. A, without cross-linker; B, Cross-linked with DMS; C, cross-linked with EGS. No add = apoprotein without DTT or metal.

protein so that some Lys or Arg residues are shielded or brought inward and results in decrease of overall basic charge on the surface, hence the faster migration toward cathode as compared to apo-protein. The fact that more dimer was obtained when EGS was used, as compared to DMS, indicated that the nearest distance between the Lys residues from each subunit must be slightly more than 11 Å, and up to at least 16 Å. Although the results proved that Cd(II)-ATOX1 and, to some extent, Hg(II)-ATOX1 complexes existed as dimers, it did not exclude the possibility that the other metal-complexes were also dimers. Specifically, apo-ATOX1, as well as other metal-ATOX1 proteins, may exist as dimers but have different conformations from that of Cd(II)-ATOX1 in such a way that the Lys residues from each subunit are too far apart to be cross-linked. An X-ray crystallographic study of metal-ATOX1 complexes showed that Cd(II) ligated to ATOX1 in a perfect tetrahedral configuration by sharing 4 Cys residues between two subunits /39/. Our results confirmed that Cd(II)-ATOX1 exists as a dimer. Regarding Cu(I) and Hg(II) bindings, the results from Wernimont *et al* /39/ showed that these two metals could also ligate ATOX1 as a dimer through a distorted tetrahedral configuration. With respect to Cu(I)-ATOX1, according to their proposed mechanism for metal transfer between subunits; at equilibrium there would be a mixture of the copper-bound forms as a monomer (when Cu(I) bound digonally between two essential Cys residues on the same subunit), and as a dimer (when Cu bound 3 or 4 Cys residues trigonally or tetrahedrally between two subunits). The reason why we could not detect more dimer form of Cu(I)-ATOX1 than that of apo-protein by cross-linking reaction might be explained by the lack of stability of Cu(I)-ATOX1 dimer in solution.

### Characterization of metal-complexes with ATOX1 mutants and with yeast Atx1

Unlike ATOX1, the NMR, and X-ray crystallographic studies of Atx1 showed that the yeast protein formed complexes with Cu(I) and Hg(II) as monomer. In general, Atx1 and ATOX1 are very similar except that Atx1 is five amino acids longer, and there are only two Cys residues in Atx1 while ATOX1 has three residues. Given the possibility that the extra Cys residue ($C^{41}$) in ATOX1 plays a role in dimerization, we mutated this Cys residue to Ser. In addition, we generated four other mutants of ATOX1, which have conserved Cys residues, mutated to Ser, either singly or in various combinations as described in the Materials and Methods section. The first four mutants listed could be expressed in large quantities similar to the wild type. They were further characterized for their metal binding capability by comparing gel mobility on native gel as criteria for binding. However, for unknown reasons, cells bearing CC/CS-ATOX1 plasmid could not express the protein. Results shown in Figure 3 indicate that the three mutants lacking one or both of conserved Cys residues, namely CC/SC-, CC/SS- and 3C/3S-ATOX1, behaved differently from wild type. The presence of DTT alone or together with metal did not alter gel migration patterns from that of untreated apo-proteins. However, C41S-ATOX1 behaved in a similar fashion to wild type in response to reduction and to the presence of metals. Cross-linking experiments also showed dimerization of C41S-ATOX1 in the presence of Cd(II), similar to what was observed with the wild type protein (data not shown). Since this mutant was generated to imitate Atx1, this observation implied that Atx1 should bind to various metals and behave in the same fashion as ATOX1 as well.

To prove this point, the yeast Atx1 was cloned, expressed, purified. and its metal-complexes were characterized. As expected, Atx1 behaved identically to ATOX1 in all aspects (Figure 4). Its tendency to

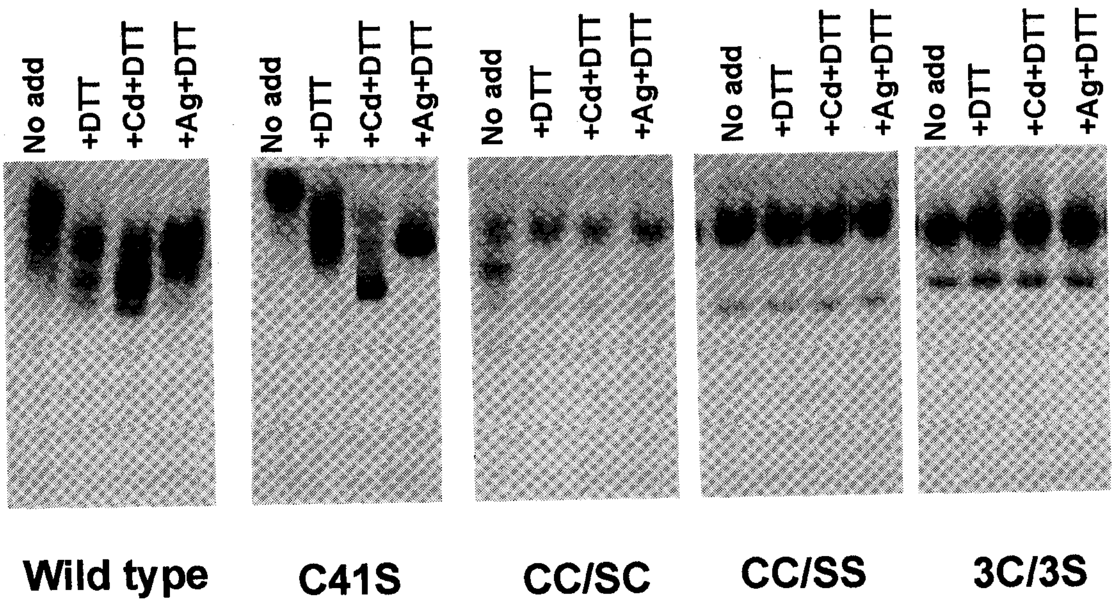

**Fig. 3:** Comparison of migration pattern on Native-PAGE of Wild-type and various mutants of ATOX1 with and without metals. C41S, non-conserved Cys ($C^{41}$) was mutated; CC/SS, both of conserved Cys ($C^{12}$ and $C^{15}$) were mutated; 3C/3S, all 3 Cys residues were mutated; CC/SC, only one of conserved Cys ($C^{12}$) was mutated. No add = apoprotein without DTT or metal.

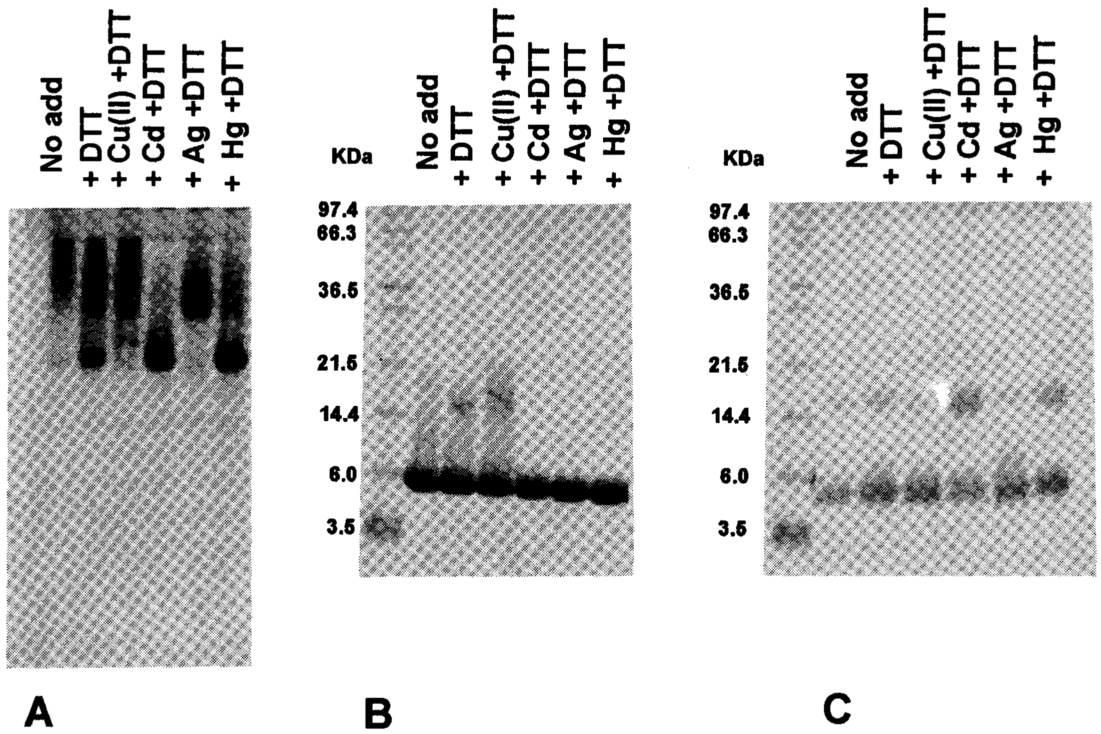

**Fig. 4:** Characterization of apo and metal-Atx1 complexes by gel electrophoresis. A, 10% Native-PAGE; B, 16% SDS-PAGE without 2-mercaptoethanol; C, 16% SDS-PAGE (without 2-mercaptoethanol) of cross-linked product of Atx1 and EGS in the presence of metals. No add = apoprotein without DTT or metal.

dimerize in the presence of both Cd(II) as well as Hg(II) (Figure 4C) is different from the observation from crystal structure of Hg(II)-Atx1 which found the metal complex to be a monomer /33/. From these findings, it is apparent that ATOX1 and Atx1 bind metals in similar manners. Based on the cross-linking experiments there is no question that in the presence of Cd(II) and Hg(II) both proteins exist as dimers. However, whether Cu(I)-, Ag(I)- or apoprotein exists as monomer or dimer remains unclear.

### Size exclusion chromatography of ATOX1, Atx1 and their metal-complexes

ATOX1, C41S-ATOX1, and Atx1, under various conditions such as untreated (oxidized apoprotein), reduced apoprotein and Cu(I)-, Ag(I)-, or Cd-complex., were characterized on a Bio-Gel P-10 column. The concentration of proteins used was 0.3, 0.5 and 0.3 mM for ATOX1, C41S-ATOX1 and Atx1 respectively. The concentration of metals and DTT used was at 1:1 and 3:1 molar equivalent of protein, respectively. The elution profiles of these complexes are presented in Figure 5, which shows profiles of the apoprotein and metal-complexes of Atx1 as an example. In general, the elution profiles of the three proteins are similar in many aspects. The oxidized apoproteins were eluted with a peak at fraction 39 - 40 (fraction size of 1 ml). Cu(I)- or Ag(I)-complex was also eluted off at a similar location. However, the reduced apoproteins as well

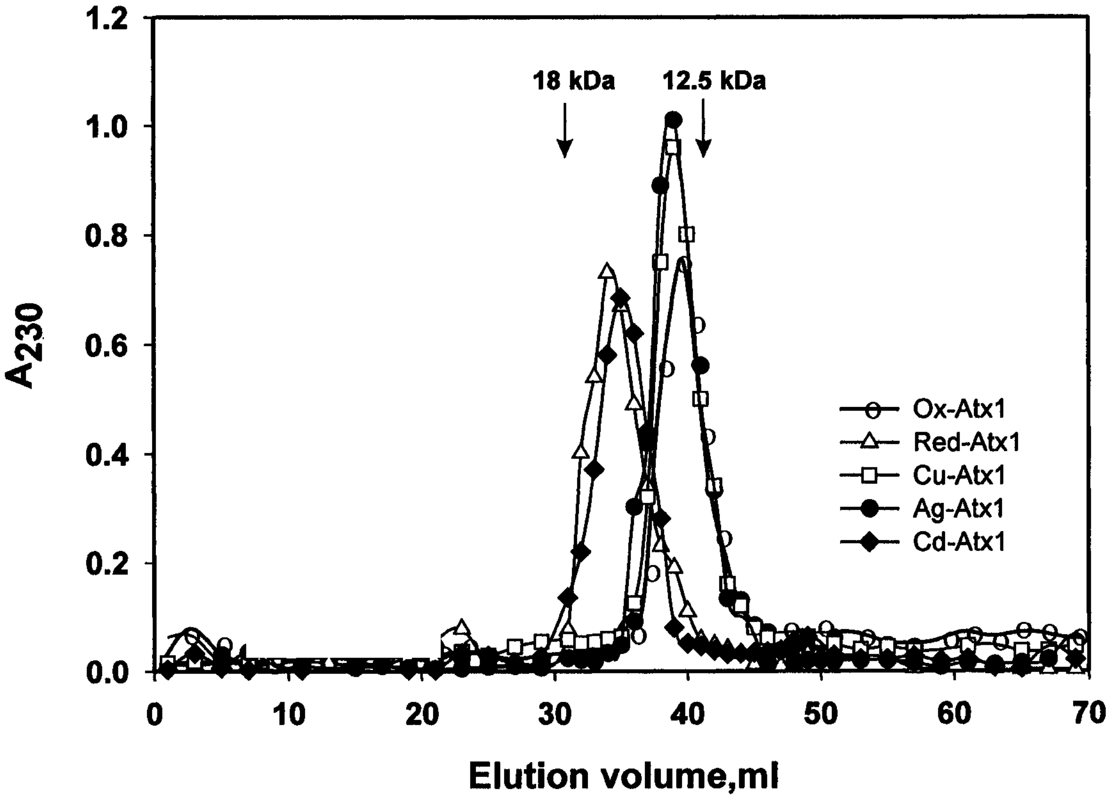

**Fig. 5:** Size Exclusion chromatography of Atx1. 0.5 ml of 0.3 mM samples of untreated apo-Atx1 (oxidized), or reduced-Atx1 (apoprotein+DTT), or metal-Atx1 complexes (apoprotein +metals +DTT) were loaded onto a Bio-Gel P-10 column (1.5x60 cm). One-ml fractions were collected. Metal and DTT were added at 1:1 and 3:1 molar ratio respectively relative to protein concentration. $V_0$ and $V_t$ of the column are at fraction 28 and 68 respectively. The elusion positions of myoglobin (18 kDa) and cytochrome C (12.5 kDa) are marked by the arrows.

as Cd(II)-complex were eluted off earlier with a peak at fraction 34-35. When compared with elution profiles of standard molecular weight proteins; myoglobin (18 kDa) and cytochrome C (12.5 kDa); the oxidized apoprotein and Cu(I)-or Ag(I)-complex have a molecular weight slightly larger than 12.5 kDa whereas the reduced apoprotein and Cd(II)-complex are slightly smaller than 18 kDa but larger than oxidized apoprotein and Cu(I)- or Ag(I)-complex. The molecular weights of ATOX1 and Atx1 subunit are 7.813 kDa and 8.518 kDa respectively. Thus it is likely that the reduced-apoprotein and the Cd(II)-complex are dimers. It was surprising that the reduced apoprotein emerged as a dimer. Obviously dimerization involved hydrophobic interaction or some form of interaction other than disulfide formation. As for the oxidized apoprotein, Cu(I)- or Ag(I)-complex, their elution profiles fit neither as a monomer nor a dimer. One possible explanation is that the peak may consist of the mixture of monomer and dimer. When the Cu(I) and Cd(II) content in the peak fraction of protein complexes was determined, there was only about 0.5-0.6 mol of Cu(I) detected per mol of protein, but 1 mol of Cd(II) was found per mol of protein. If the Cu(I) and Cd(II)-complexes are formed with ATOX1 or Atx1 by sharing –SH groups between two subunits, then the expected amount of metal incorporated should be 0.5 mol/mol protein. Cu(I) content obtained from Cu(I)-Atx1 peak seems to fit this assumption. At equilibrium there may be a mixture of Cu(I)-Atx1 dimers where two subunits share one bound Cu(I), some monomers with one mol of Cu(I) bound per subunit via digonal coordination, as well as some metal free subunits. As for Cd(II)-Atx1, although the physical evidence strongly suggests that the protein exists as a dimer, the 1:1 ratio of metal:protein does not support two subunits sharing one Cd(II) as seen in X-ray crystal structure /39/. Based on our results, the plausible explanation would be either Cd(II) bound to Atx1 as 1:1 ratio via digonal coordinate or the protein dimers share two mol of Cd(II) in a dimetal cluster

When ATOX1 was incubated with 2-fold excess of Cu(II) plus DTT and at higher protein concentration (1 mM), the elution profile of Cu(I)-complex emerged as two well-separated peaks (Figure 6). The first peak came off at the void volume and contained some precipitation; the second peak was at fraction 35-36, which is the range of dimer/monomer mixture. The first peak contained some aggregated Cu(I)-bound protein, which formed a yellowish-green precipitate after centrifugation. The Cu(I) content in the soluble part of this peak was about 2 mol per mol of protein, whereas the Cu(I) content in the second peak was only 0.5 per mol. The precipitation of Cu(I)-protein complex, when ATOX1 was used at high concentration and in the presence of 2 times excess copper, has been consistently observed in our laboratory when the dialysis method was used to remove excess copper, even under inert atmosphere of argon gas. On all occasions, although the solution of the protein-Cu mixture was clear during the incubation period (30 min), a significant amount of

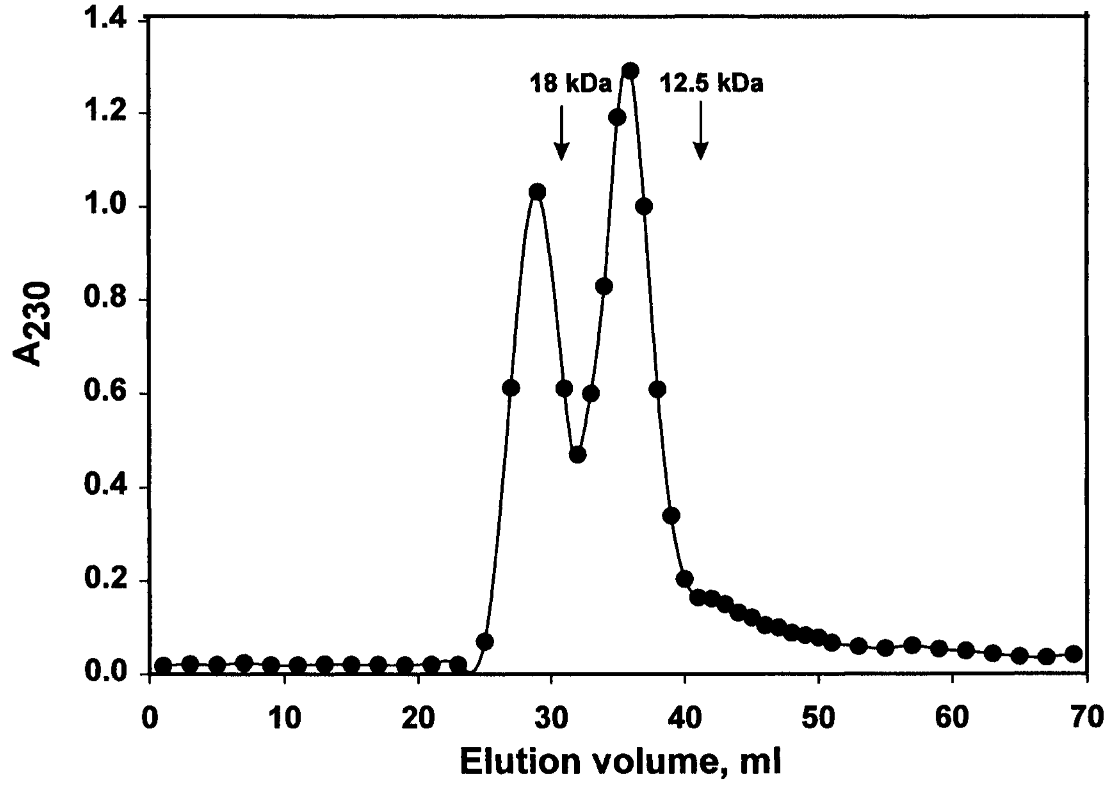

**Fig. 6:** Size Exclusion chromatography of Cu(I)-ATOX1. 0.5 ml of 1 mM Atox1 +2 mM Cu(II)+3 mM DTT was loaded onto a Bio-Gel P-10 column. All conditions are the same as in Figure 5

protein precipitated during the dialysis period, and the precipitate always had a yellowish-green color. Initially we suspected that the precipitation might be due to the presence of excess Cu(II) adversely affecting the protein, because dialysis did not remove it fast enough to prevent this consequence. In contrast, in the gel filtration experiments, we expected that the protein complex would be separated from the excess Cu(II) faster than by dialysis and precipitation would not take place. It should be pointed out that at the time of loading to the column, the protein mixture did not show any sign of precipitation; nevertheless, the first peak of Cu(I)-ATOX1 emerged as a mixture of insoluble and soluble oligomers, which also incorporated more Cu(I) than the complex in the second peak. These findings suggest that polymerization of Cu(I)-ATOX1 depends on protein as well as copper concentrations. A similar observation on Cu(I)-CopZ has also been reported /36/: that Cu(I)-protein complex aggregated at protein concentration higher than 0.7 mM. The oligomeric state of Cu(I)-ATOX1 contains at least 2 mol of Cu(I) per mol of protein. The copper chaperone protein Cox 17, the mitochondrial metallochaperone, has been reported to exist as a dimer/tetramer of polycopper complex, with each subunit binding 3 mol of Cu(I) as a trinuclear cluster /44/. Copper was also reported to enhance homodimerization of yeast CCS, a copper chaperone for SOD1 (*45*). Domains I and III of hCCS, a human homologue, were found to interact with each other via cysteine-bridged dicopper cluster, with up to 3.5 mol of Cu(I) bound per mol /46/. How multiple Cu(I) atoms bind to ATOX1 oligomers is unclear and further investigation is currently underway.

### UV-visible spectral characteristics of metal-protein complexes of Atxl and ATOX1

The peak fraction of metal-protein complexes of Atx1, obtained from gel filtration experiments shown in Figure 5, were scanned between the wavelengths of 240-360 nm. Other than the protein peak at 276 nm, all metal-bound proteins displayed an increase in absorbance at 254 nm, as compared to apoprotein, but with different intensity depending on the metal used. Ag(I)-complex was the highest, followed by Cu(I)-complex, then Cd(II)-complex, which had the weakest absorption at this wavelength (Figure 7). The scans of ATOX1 or C41S-ATOX1 complexed with metals also showed similar results (data not shown). The absorbance at 254 nm is probably due to the metal thiolate bond of metal protein complex.

Next, a titration experiment was attempted by following the increase in the absorbance at 254 nm after addition of Cu(II) solution to the freshly reduced ATOX1. Figure 8A shows typical results of titration between reduced ATOX1 with increasing concentration of Cu(II), from 0.1 to 6 molar ratios with respect to protein. As can be seen, the absorbance at 254 nm increased with increasing ratio of Cu(II) and slowly leveled off after 2:1 molar ratio of Cu(II) to protein was added. After ATOX1 was titrated with 6 molar excess of Cu(II), there was only a total of 1 mol of Cu(I) incorporated per mol of protein, as determined by BCS reaction with the metal-protein mixture. BCS reacts specifically with Cu(I) and can be used to assay Cu(I) in the presence of excess Cu(II) (*43*) The Cu(I)-ATOX1 complex (or CuATOX1) was relatively stable for at least 5 days. It should be pointed out also that these experiments were performed under normal laboratory conditions without any extra precaution of maintaining anaerobic environment. Under these conditions, there was no evidence of precipitation when excess Cu(II) was used. These findings contrasted the result obtained when the protein was used at 1 mM and with 2x Cu(II), which resulted in two species of Cu(I)-complexes, one more aggregated containing 2 mol of Cu(I)/mol protein, the other soluble but

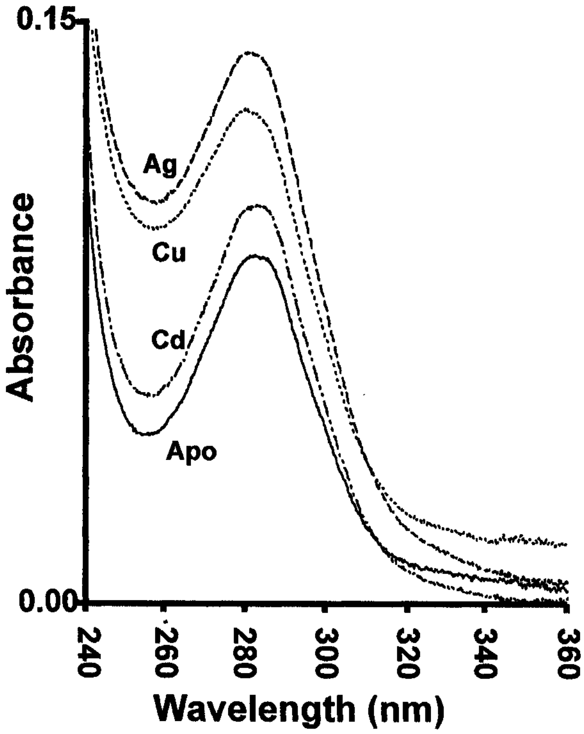

**Fig. 7:** UV-visible Spectra of metal-Atx1 complexes. Peak fractions (15 μM, each) from reduced apo-Atx1, Cd(II)-Atx1, Cu(I)-Atx1 and Ag(I)-Atx1 as shown in Figure 5 were scanned between 240-360 nm containing only 0.5 mol of Cu(I) (see Figure 6). From titration experiments, due to low protein concentration (15 μM), it was not possible to identify whether there was one or two species of complexes in the solution. The presence of Cu(I) at 1 mol/mol may represent the homogeneous species of Cu(I) bound in digonal geometry or it may consist of the mixture of dicopper and half-copper species as observed in Figure 6.

Titration of reduced ATOX1 with Ag(I) also generated a pattern of incremental absorbance at 254 nm that was almost identical to that of Cu(II) titration (Figure 8B). The increase of absorbance generated by Cu(II) or Ag(I) titration was reversible after the pH of the mixture was decreased below pH 2. Titration with Cd(II) produced a very small increase in absorbance at 254 nm (data not shown).

When oxidized ATOX1 was titrated with Cu(II), surprisingly, there was also an increase in absorbance at 254 nm, which reached saturation with excess Cu(II) (Figure 8C). However, unlike the reduced protein, the oxidized ATOX1 did not have any interaction with Ag(I) since there was no increase in absorbance at 254 nm with addition of Ag(I) (Figure 8D). There was also a pronounced difference in absorbance of Cu-thiolate products of oxidized and reduced ATOX1 at wavelength 300 nm (see Figures 8A and 8C). There was only 0.2-0.25 mol of Cu(I) formed per mol of protein, in the mixture of oxidized ATOX1 and 6 molar excess of Cu(II), as opposed to 1:1 mol ratio when reduced protein was used. One possible argument for finding some Cu(I)–complex with oxidized protein is that there was still a substantial residual amount of reduced form in the mixture of "oxidized protein"; however, the lack of response to Ag(I) titration does not support this explanation. When oxidized ATOX1 (0.89 mM) was incubated with 3 molar excess of Cu(II) and then separated on a gel filtration column, aggregation of protein was not observed, as was the case when ATOX1

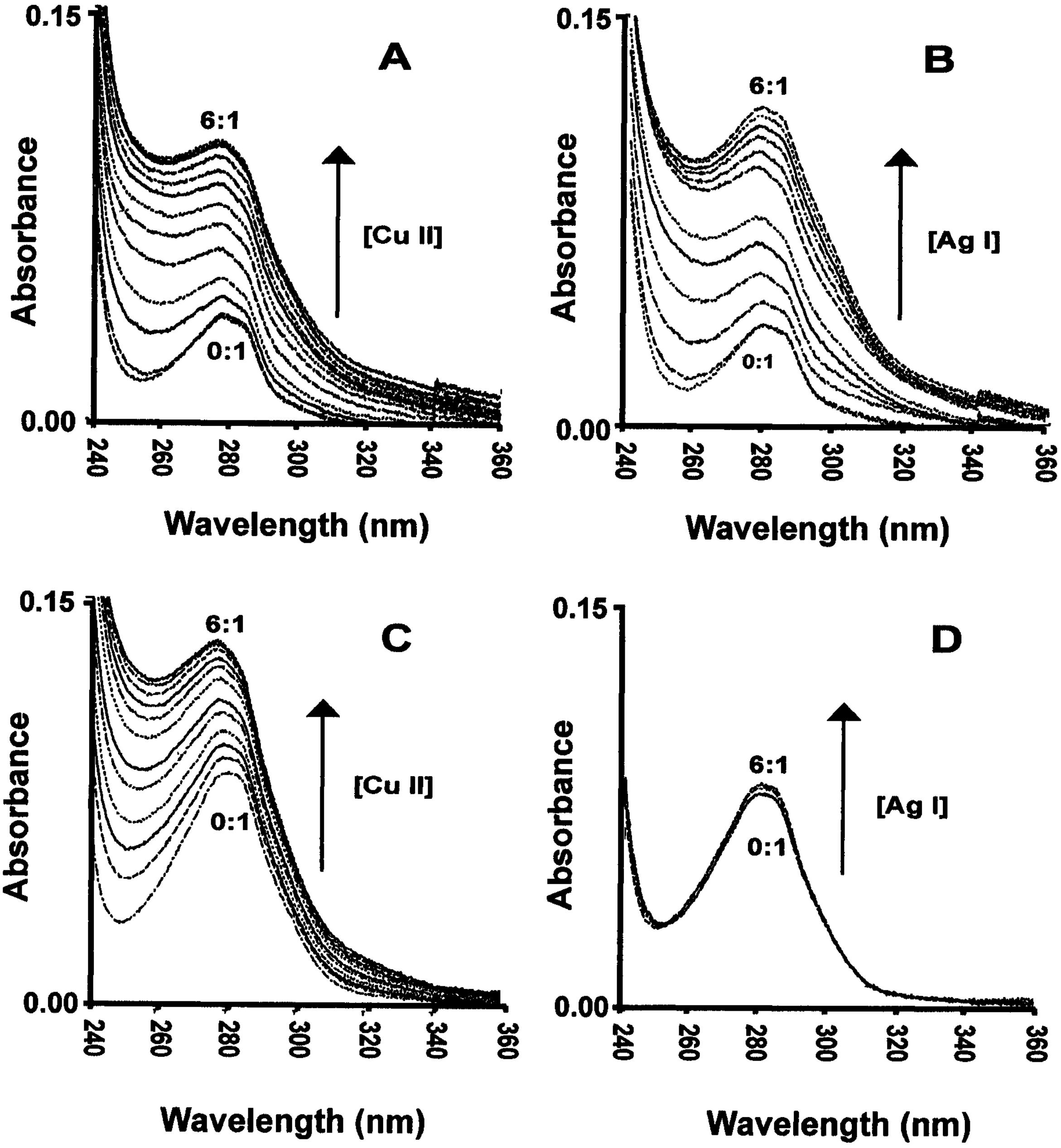

**Fig. 8:** UV-visible Spectra of the titration of Reduced and Oxidized ATOX1 with Cu(II) and Ag(I). 15 μM of oxidized or freshly reduced ATOX1 was titrated with small increment of $CuSO_4$ or $AgNO_3$. Metals were added at 0, 0.1, 0.2, 0.4, 0.6, 0.8, 1.0, 1.5, 2.0, 2.5, 3.0, 4.0, 5.0 and 6.0 molar ratio with respect to protein concentration. The arrows indicate the direction of spectra from each addition. A, Reduced ATOX1 + $CuSO_4$. B, Reduced ATOX1 + $AgNO_3$. C, Oxidized ATOX1 + $CuSO_4$. D, Oxidized ATOX1 + $AgNO_3$.

was incubated with 2:1 Cu(II) in the presence of DTT, and then separated on the column (shown in Figure 6). After treatment, the protein still emerged at the same position as untreated oxidized ATOX1. Furthermore, neither Cu(I) nor Cu(II) was detected in the protein fractions. When an aliquot from these fractions was retitrated with Cu(II) and Ag(I), it produced the same results shown in Figure 8 C and D, with a yield of Cu(I) formation of 0.2 mol per mol of protein. These findings suggest that both oxidized and reduced forms of ATOX1 are able to bind Cu(II) and convert it to Cu(I) but the capacity for reduction of Cu(II) is much more limited in the oxidized form. Additionally, the affinity of oxidized ATOX1 for copper is much weaker, and the binding is unstable as compared to the reduced protein. The reduction of the protein and its subsequent dimerization may be a prerequisite for metal binding and its stabilization. To our knowledge, this is the first time that Atx1 or ATOX1 was demonstrated to bind Cu(II) and reduce it to Cu(I) without an exogenous reducing agent being present. Previously, Cu(I)-protein complexes of various copper chaperones and of the copper-binding domains of Wilson and Menkes proteins were obtained either by adding Cu(II) plus excess reducing agent /21,31/37,47/, or Cu(I) plus reducing agent /36,45,48/ to the protein solution. As for Ag(I), this metal could bind only to the reduced ATOX1 but not to the oxidized form, if the increase in absorbance at 254 nm is taken as a criterion for binding.

To clarify the differences between reduced and oxidized ATOX1 and their respective metal complexes further, these samples were subjected to native gel electrophoresis. As shown in Figure 9A, the oxidized protein consisted of two bands with equal intensity and addition of Cu(II), Ag(I) or Cd(II) had no effect on migration pattern of the protein. However, there were striking differences among products of metal complexes of freshly reduced protein, as shown in Figure 9B. Compared to the oxidized form, which contained two bands, the reduced protein consisted mainly of a slower migrating upper band. Cu(II) did not change the migration pattern from that of reduced protein alone, but the addition of an identical amount of Ag(I) resulted in a complex which migrated faster than the apoprotein. This occurred in spite of the fact that both metals produced a similar increase in absorbance at 254 nm. Since Cu(I) and Ag(I) have identical

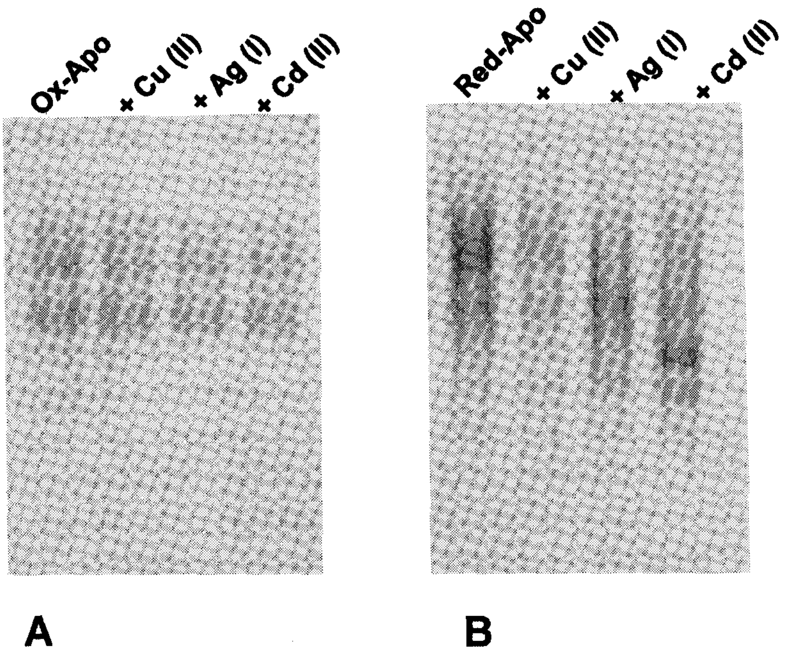

**Fig. 9:** Native-PAGE of metal complexes formed by direct addition (1:1 mol ratio) of Cu (II), Ag (I) or Cd (II) to oxidized, (A); or freshly reduced ATOX1, (B).

charges, if both metals bind to the protein with the same coordination, then the overall charge effect on the complex should be the same. The difference in migration patterns on native-PAGE suggests that complexes formed by these two metals with ATOX1 are different.

Ag(I) was used as a ligating metal in an NMR study of the 4$^{th}$ Cu-binding domain of Menkes protein /35/; it formed a complex with the Cys residue in the MXCXXC motif of this peptide in a digonal geometry. Cu(I) and Ag(I) were assumed to coordinate this motif in a similar fashion. Radioactive silver has been used in fibroblast silver loading for diagnosis of Menkes disease /49/. Beside Cu(I), Ag(I) is also a substrate for a bacterial /50/, as well as a yeast /51/, P-type ATPase homologue of Wilson and Menkes protein. The Cu/Ag-ATPase, CopB, in *E. hirae* was found to have the same apparent Km for both metals implying similarity in metal binding /50/. However, the results from our study suggest that Ag(I) and Cu(I) binding to ATOX1 and Atx1 cause different changes in conformation of the proteins.

Binding of Cd(II) to reduced Atox1 also resulted in a complex that migrated faster than Ag(I)-complex. In this experiment, the patterns of gel migration of metal complexes formed by direct addition of metals to the reduced protein in the absence of DTT are similar to those formed in the presence of DTT as shown in Figure 1A.

### CONCLUSIONS

We have presented evidence that ATOX1 and Atx1, as expressed, purified and used in this study, are indistinguishable from each other in terms of subunit structure and metal binding properties. Both proteins show the ability to bind to various heavy metals such as Cu(I), Ag(I), Cd(II) and Hg(II). However, irrespective of whether ATOX1 or Atx1 is used, each of these metals seems to confer different effects on the conformation or oligomerization state of the protein, as demonstrated by migration patterns on native gel electrophoresis, by cross-linking reactions and by different intensity of absorbance at 254 nm, which represent metal-thiolate charge transfer spectra. Moreover, we are able to show that ATOX1 can bind and reduce Cu(II) into Cu(I) in the absence of added reducing agent. Thus the protein indeed possesses redox activity. Previously, the Cu(I) complex of ATOX1, Atx1, or copper-binding motif of Menkes or Wilson disease protein was only demonstrated by addition of either Cu(I) solution to the reduced protein or by adding Cu(II) solution in the presence of excess reducing agent to the protein solution. In this study, freshly reduced ATOX1 was able to reduce Cu(II) to Cu(I) stoichiometrically to mol equivalent of the protein present irrespective of the excess amount of Cu(II) added. The oxidized protein was also able to convert Cu(II) to Cu(I) but with only about 20% efficiency, although in both cases there were similar increases in absorbance at 254 nm after same amount of Cu(II) was added. Unlike Cu(II), Ag(I) was able to bind only to the reduced form of the protein, and although Ag(I)-ATOX1 displayed similar increase in UV-visible spectra as Cu(I)-ATOX1, their conformations were definitely different as shown by native gel migration patterns.

Our studies provide strong evidence that Cd(II)-complex of either protein is a dimer, the Hg(II)-complex is also dimeric, but Cu(I)-complex is a mixture of various forms depending on the concentration of protein and the molar ratio of copper used. These findings indicate the need for cautious interpretation of structural data for the copper chaperones or their target proteins. In the past, Ag(I), Hg(II), Cu(I) or Cd(II) has been used in NMR or X-ray crystallographic studies of the structure of these proteins /33-36,39/. Ag(I) was used as a ligating metal in NMR study of the fourth copper binding domain of Menkes protein /35/. In the case of ATOX1, the crystal structure of Cd(II), Hg(II) as well as Cu(I)-complex revealed the presence of dimer for all three metal complexes /39/, whereas Hg (II)-Atx1 /33/ or Cu(I)-Atx1 /34/ was found to exist as a monomer. Although Ag(I) had never been used in the studies of either ATOX1 or Atx1, from our studies presented here it is clear that Ag(I)- complex of both proteins is different from that of Cu(I)-complex.

### ACKNOWLEDGMENT

We thank Drs. Diane W. Cox and Valeria C. Culotta for cDNAs of ATOX1 and Atx1 respectively. We also thank Dr. Michael DiDonato for his constructive comments.

### REFERENCES

1. C. D. Vulpe and S. Packman, *Annu Rev Nutr* **15**, 293 (1995).
2. C. Vulpe, B. Levinson, S. Whitney, S. Packman, and J. Gitschier, *Nat Genet* **3**, 7 (1993).
3. J. F. Mercer, J. Livingston, B. Hall, J. A. Paynter, C. Begy, S. Chandrasekharappa, P. Lockhart, A. Grimes, M. Bhave, D. Siemieniak, and et al., *Nat Genet* **3**, 20 (1993).
4. P. C. Bull, G. R. Thomas, J. M. Rommens, J. R. Forbes, and D. W. Cox, *Nat Genet* **5**, 327 (1993).
5. R. E. Tanzi, K. Petrukhin, I. Chernov, J. L. Pellequer, W. Wasco, B. Ross, D. M. Romano, E. Parano, L. Pavone, L. M. Brzustowicz, and et al., *Nat Genet* **5**, 344 (1993).
6. Y. Yamaguchi, M. E. Heiny, M. Suzuki, and J. D. Gitlin, *Proc Natl Acad Sci U S A* **93**, 14030 (1996).
7. M. J. Petris, J. F. Mercer, J. G. Culvenor, P. Lockhart, P. A. Gleeson, and J. Camakaris, *Embo J* **15**, 6084 (1996).
8. H. A. Dierick, A. N. Adam, J. F. Escara-Wilke, and T. W. Glover, *Hum Mol Genet* **6**, 409 (1997).
9. I. H. Hung, M. Suzuki, Y. Yamaguchi, D. S. Yuan, R. D. Klausner, and J. D. Gitlin, *J Biol Chem* **272**, 21461 (1997).
10. X. L. Yang, N. Miura, Y. Kawarada, K. Terada, K. Petrukhin, T. Gilliam, and T. Sugiyama, *Biochem J* **326**, 897 (1997).
11. B. Sarkar, *Chem Rev* **99**, 2535 (1999).
12. D. S. Yuan, R. Stearman, A. Dancis, T. Dunn, T. Beeler, and R. D. Klausner, *Proc Natl Acad Sci U S A* **92**, 2632 (1995).
13. S. J. Lin and V. C. Culotta, *Proc Natl Acad Sci U S A* **92**, 3784 (1995).
14. P. C. Bull and D. W. Cox, *Trends Genet* **10**, 246 (1994).
15. S. J. Lin, R. A. Pufahl, A. Dancis, T. V. O'Halloran, and V. C. Culotta, *J Biol Chem* **272**, 9215 (1997).
16. V. C. Culotta, L. W. Klomp, J. Strain, R. L. Casareno, B. Krems, and J. D. Gitlin, *J Biol Chem* **272**, 23469 (1997).

17. P. Cobine, W. A. Wickramasinghe, M. D. Harrison, T. Weber, M. Solioz, and C. T. Dameron, *FEBS Lett* **445**, 27 (1999).
18. C. Rensing, B. Mitra, and B. P. Rosen, *Proc Natl Acad Sci U S A* **94**, 14326 (1997).
19. C. Rensing, B. Fan, R. Sharma, B. Mitra, and B. P. Rosen, *Proc Natl Acad Sci U S A* **97**, 652 (2000).
20. C. Rensing, Y. Sun, B. Mitra, and B. P. Rosen, *J Biol Chem* **273**, 32614 (1998).
21. R. A. Pufahl, C. P. Singer, K. L. Peariso, S. J. Lin, P. J. Schmidt, C. J. Fahrni, V. C. Culotta, J. E. Penner-Hahn, and T. V. O'Halloran, *Science* **278**, 853 (1997).
22. D. L. Huffman and T. V. O'Halloran, *J Biol Chem* **275**, 18611 (2000).
23. L. W. Klomp, S. J. Lin, D. S. Yuan, R. D. Klausner, V. C. Culotta, and J. D. Gitlin, *J Biol Chem* **272**, 9221 (1997).
24. G. S. Naeve, A. M. Vana, J. R. Eggold, G. S. Kelner, R. Maki, E. B. Desouza, and A. C. Foster, *Neuroscience* **93**, 1179 (1999).
25. M. S. Nanji and D. W. Cox, *Genomics* **62**, 108 (1999).
26. P. J. Lockhart and J. F. Mercer, *Biochim Biophys Acta* **1490**, 11 (2000).
27. I. Hamza, L. W. Klomp, R. Gaedigk, R. A. White, and J. D. Gitlin, *Genomics* **63**, 294 (2000).
28. I. H. Hung, R. L. Casareno, G. Labesse, F. S. Mathews, and J. D. Gitlin, *J Biol Chem* **273**, 1749 (1998).
29. D. Larin, C. Mekios, K. Das, B. Ross, A. S. Yang, and T. C. Gilliam, *J Biol Chem* **274**, 28497 (1999).
30. I. Hamza, M. Schaefer, L. W. Klomp, and J. D. Gitlin, *Proc Natl Acad Sci U S A* **96**, 13363 (1999).
31. M. DiDonato, H. F. Hsu, S. Narindrasorasak, L. Que, Jr., and B. Sarkar, *Biochemistry* **39**, 1890 (2000).
32. M. Ralle, M. J. Cooper, S. Lutsenko, and N. J. Blackburn, *J. Am. Chem. Soc.* **120**, 13525 (1998).
33. A. C. Rosenzweig, D. L. Huffman, M. Y. Hou, A. K. Wernimont, R. A. Pufahl, and T. V. O'Halloran, *Structure Fold Des* **7**, 605 (1999).
34. F. Arnesano, L. Banci, I. Bertini, D. L. Huffman, and T. V. O'Halloran, *Biochemistry* **40**, 1528 (2001).
35. J. Gitschier, B. Moffat, D. Reilly, W. I. Wood, and W. J. Fairbrother, *Nat Struct Biol* **5**, 47 (1998).
36. R. Wimmer, T. Herrmann, M. Solioz, and K. Wuthrich, *J Biol Chem* **274**, 22597 (1999).
37. L. Banci, I. Bertini, R. Del Conte, J. Markey, and F. J. Ruiz-Duenas, *Biochemistry* **40**, 15660 (2001).
38. R. A. Steele and S. J. Opella, *Biochemistry* **36**, 6885 (1997).
39. A. K. Wernimont, D. L. Huffman, A. L. Lamb, T. V. O'Halloran, and A. C. Rosenzweig, *Nat Struct Biol* **7**, 766 (2000).
40. T. E. Creighton, "Disulfide bonds between cysteines.," p. 155. IRL Press at Oxford University Press, Oxford, 1993.
41. M. Casadevall and B. Sarkar, *J Inorg Biochem* **71**, 147 (1998).
42. A. J. Brenner and E. D. Harris, *Anal Biochem* **226**, 80 (1995).
43. J. M. Rifkind, L. D. Lauer, S. C. Chiang, and N. C. Li, *Biochemistry* **15**, 5337 (1976).
44. D. N. Heaton, G. N. George, G. Garrison, and D. R. Winge, *Biochemistry* **40**, 743 (2001).
45. P. J. Schmidt, T. D. Rae, R. A. Pufahl, T. Hamma, J. Strain, T. V. O'Halloran, and V. C. Culotta, *J Biol Chem* **274**, 23719 (1999).
46. J. F. Eisses, J. P. Stasser, M. Ralle, J. H. Kaplan, and N. J. Blackburn, *Biochemistry* **39**, 7337 (2000).
47. P. Y. Jensen, N. Bonander, N. Horn, Z. Tumer, and O. Farver, *Eur J Biochem* **264**, 890 (1999).
48. A. L. Lamb, A. S. Torres, T. V. O'Halloran, and A. C. Rosenzweig, *Biochemistry* **39**, 14720 (2000).

49. F. W. Verheijen, C. E. Beerens, A. C. Havelaar, W. J. Kleijer, and G. M. Mancini, *J Med Genet* **35**, 849 (1998).
50. M. Solioz and A. Odermatt, *J Biol Chem* **270**, 9217 (1995).
51. P. J. Riggle and C. A. Kumamoto, *J Bacteriol* **182**, 4899 (2000).

